# Supplementary material for: Tensile Behaviors and Mechanical Property Analyses of T-Welded Joint for Thin-Walled Parts in Consideration of Different TIG Welding Currents Using Multiple Damage Models and Fracture Criterions: Numerical Simulation and Experiment Validation
Source: Materials (Basel). 2023 Jul 6;16(13):4864. doi: 10.3390/ma16134864 (PMC10343321; doi:10.3390/ma16134864)
Supplement: Supplementary file 1 [file materials-16-04864-s001.zip › materials-2423518-supplementary.pdf]

Supplementary Materials—Tables in this article

Table S1 — The relative parameters for Swift model

Parameters	100A	140A	170A	220A
ϕ	351.10084	398.22921	395.24122	380.28744
ε_s	0.00396	0.00434	0.0042	0.00446
n	0.19942	0.26991	0.26161	0.23757

Table S2 — The relative parameters for Voce model

Parameters	100A	140A	170A	220A
σ_s	139.5	100.5	97.5	112.5
ψ	130.56049	148.07678	138.2901	126.71723
m	10.66971	16.02394	20.9583	20.00705

Table S3 — The relative parameters for H-S model

Parameters	100A	140A	170A	220A
σ_s	139.5	100.5	97.5	112.5
ξ	135.46196	168.21967	162.22546	139.10268
k	9.48236	8.52857	8.87108	11.86186
n	0.97722	0.85465	0.80958	0.88281

Table S4 — The relative parameters for Swift-Voce combination model (λ=0.4)

Parameters	100A	140A	170A	220A
ϕ	186.31395	579.09124	779.25954	607.35475
ε_s	0.00396	0.00434	0.0042	0.00446
n	0.059003	0.33659	0.40033	0.33257
ψ	206.09922	96.86013	56.66304	70.45496
m	9.56423	18.23885	45.01308	28.38472

Table S5 The relative parameters for Swift-Voce combination model (λ=0.6)

Parameters	100A	140A	170A	220A
ϕ	169.17534	395.94388	537.25610	423.10899
ε_s	0.00396	0.00434	0.0042	0.00446
n	0.03937	0.26665	0.32950	0.26213
ψ	310.42655	147.35838	66.41993	99.51346
m	9.55250	16.00958	43.38825	24.97682

Table S6 — The relative parameters for Swift-Voce combination model (λ=0.8)

Parameters	100A	140A	170A	220A
ϕ	161.20751	306.65505	418.64691	334.61802
ε_s	0.00396	0.00434	0.0042	0.00446
n	0.02954	0.21845	0.28144	0.21633
ψ	622.08267	309.78983	109.91816	197.30807
m	9.54726	14.51861	39.34351	22.41282

Table S7 — The parameter value for Voce-H-S combination model (λ=0.4)

Parameters	100A	140A	170A	220A
ξ	278.00539	30.64830	45.98356	29.00182
k	16.19586	1083922.178	261856.5118	63242.74685
n	1.18829	2.93917	2.61421	2.37963
ψ	22.34592	244.67361	225.02909	207.70637
m	61.92545	12.57749	14.39272	15.50604

Table S8 — The parameter value for Voce-H-S combination model (λ=0.6)

Parameters	100A	140A	170A	220A
ξ	185.349	20.80647	30.99811	19.33901
k	16.19307	302364.5365	149947.7498	62720.11307
n	1.18822	2.68039	2.501903	2.37793
ψ	33.50718	366.98747	337.53957	311.55901
m	61.93251	12.52141	14.32799	15.50511

Table S9 — The parameter value for Voce-H-S combination model (λ=0.8)

Parameters	100A	140A	170A	220A
ξ	139.00282	15.60268	23.24988	117.93708
k	16.19582	305109.6394	149522.9691	22.45722
n	1.18829	2.68222	2.50133	1.23831
ψ	67.03743	733.97485	675.07958	206.20270
m	61.92555	12.52195	14.32765	59.72887

Table S10 — The parameter value for Swift-H-S combination model (λ=0.4)

Parameters	100A	140A	170A	220A
ϕ	347.31059	87.18754	5.19502	47.56016
ξ	116.15585	326.87451	472.91115	317.24954
k	23.37481	5.22945	2.49436	5.34757

n_1	0.17634	0.02973	-0.22498	-0.07888
n_2	1.29688	0.71652	0.49319	0.65680

Table S11 — The parameter value for Swift-H-S combination model (λ=0.6)

Parameters	100A	140A	170A	220A
ϕ	251.47434	103.28559	9.98220	49.28079
ξ	194.52979	472.97210	760.18055	506.42908
k	20.76538	5.36841	2.42056	5.14849
n_1	0.11419	0.03890	-0.24473	-0.09882
n_2	1.26145	0.72570	0.47485	0.64144

Table S12 — The parameter value for Swift-H-S combination model (λ=0.8)

Parameters	100A	140A	170A	220A
ϕ	215.71425	121.65580	11.27898	46.34019
ξ	405.95097	872.31351	1625.98090	1090.80824
k	19.86019	5.70102	2.36030	4.93206
n_1	0.08457	0.05798	-0.26260	-0.12085
n_2	1.24774	0.74603	0.45710	0.62293

Table S13 — The correlation coefficient value for different models

Parameters	100A	140A	170A	220A	Mean value
J-C	0.996381	0.994048	0.991825	0.995960	0.994554
H-S	0.997614	0.994244	0.993187	0.997395	0.995610
Swift	0.997418	0.996362	0.994230	0.997548	0.996390
Voce	0.997654	0.993138	0.992216	0.997446	0.995114
0.2HS-V	0.99759	0.997150	0.994904	0.997496	0.996785
0.4HS-V	0.997599	0.99509	0.994882	0.997509	0.996270
0.6HS-V	0.997604	0.995103	0.994895	0.997497	0.996275
0.8HS-V	0.997591	0.995098	0.994896	0.997596	0.996295
0.2S-HS	0.997588	0.997710	0.994904	0.997884	0.997022
0.4S-HS	0.997604	0.985037	0.994882	0.997894	0.993854
0.6S-HS	0.997602	0.995776	0.994895	0.997900	0.996543
0.8S-HS	0.997592	0.995727	0.994896	0.997902	0.996529
0.2S-V	0.997688	0.99486	0.994931	0.997874	0.996338
0.4S-V	0.997679	0.995102	0.99515	0.997857	0.996447
0.6S-V	0.997670	0.995535	0.995247	0.997609	0.996515
0.8S-V	0.997673	0.99543	0.995262	0.997828	0.996548

Table S14 — The MSE value for different models

Parameters	100A	140A	170A	220A	Mean value
J-C	0.361828	0.216333	0.233216	0.052057	0.215858
H-S	0.267657	0.190173	0.209303	0.034078	0.175303
Swift	0.361738	0.216070	0.233335	0.052206	0.215837
Voce	0.361816	0.215769	0.233702	0.052493	0.215945
0.2HS-V	0.266765	0.042449	0.194651	0.037814	0.135420
0.4HS-V	0.266776	0.178807	0.195107	0.037529	0.169555
0.6HS-V	0.266777	0.178793	0.195101	0.037527	0.169550
0.8HS-V	0.266331	0.178653	0.194321	0.049037	0.172085
0.2S-HS	0.255664	0.183009	0.194651	0.049467	0.170698
0.4S-HS	0.257025	0.182941	0.195107	0.049867	0.171235
0.6S-HS	0.256796	0.183402	0.194787	0.050306	0.171323
0.8S-HS	0.256990	0.183024	0.194321	0.028845	0.165795
0.2S-V	0.331267	0.203005	0.210079	0.039760	0.196028
0.4S-V	0.330868	0.200639	0.208857	0.041675	0.195510
0.6S-V	0.330742	0.197457	0.209317	0.043804	0.195330
0.8S-V	0.330774	0.195636	0.210113	0.044213	0.195184
